# The potential of school-based WASH programming to support children as agents of change in rural Zambian households

**DOI:** 10.1186/s12889-021-11824-3

**Published:** 2021-10-08

**Authors:** James C. Winter, Gary L. Darmstadt, Samantha J. Lee, Jennifer Davis

**Affiliations:** 1 Department of Civil and Environmental Engineering, Stanford University, Yang and Yamazaki Environment and Energy Building, 473 Via Ortega, Office 161, Stanford, CA 94305 USA; 2 Department of Pediatrics, Stanford University School of Medicine, 1701 Page Mill Road, Palo Alto, CA 94304 USA; 3 Woods Institute for the Environment, Stanford University, Yang and Yamazaki Environment and Energy Building, 473 Via Ortega, Office 161, Stanford, CA 94305 USA

**Keywords:** WASH in schools, Behavior change, Agents of change, Health education, Rural, Zambia, Sub-Saharan Africa

## Abstract

**Background:**

Water, sanitation, and hygiene (WASH) interventions frequently assume that students who learn positive WASH behaviors will disseminate this information to their families. This is most prominent in school-based programs, which rely on students to act as “agents of change” to translate impact from school to home. However, there is little evidence to support or contradict this assumption.

**Methods:**

We conducted a quasi-experimental, prospective cohort study in 12 schools in rural, southern Zambia to measure the impact of WASH UP!, a school-based WASH program designed by the creators of Sesame Street. WASH UP! is an educational program that uses stories and interactive games to teach students in grades 1–4 about healthy behaviors, such as washing hands and using the latrine. We completed in-person interviews with grade 1 and 4 students (*N* = 392 and 369, respectively), their teachers (*N* = 24) and caregivers (*N* = 729) using structured surveys containing both open- and closed-ended questions. We measured changes in knowledge and whether students reported sharing WASH-related messages learned in school with their caregivers at home.

**Results:**

Student knowledge increased significantly, but primarily among students in grade 1. Overall rates of students reporting that they shared messages from the curriculum with their caregivers rose from 7 to 23% (*p* <  0.001). Students in grade 4 were 5.2 times as likely as those in grade 1 to report sharing a WASH-related message with their caregivers (ARR = 5.2, 95% C.I. = (2.3, 8.9); p <  0.001).

**Conclusions:**

Although we measured only modest levels of student dissemination of WASH UP! messages from the school to the home, students in grade 4 showed significantly more promise as agents of change than those in grade 1. Future work should prioritize developing curricula that reflect the variability in needs, capabilities and support in the home and community among primary school students rather than a single approach for a wide range of ages and contexts.

**Supplementary Information:**

The online version contains supplementary material available at 10.1186/s12889-021-11824-3.

## Introduction

Consistent practice of recommended water, sanitation, and hygiene (WASH) behaviors is central to reducing exposure to fecal contamination and improving health outcomes for children [1]. Increasing the frequency of these behaviors requires a combination of factors, including convenient access to functional WASH infrastructure and targeted behavior change messaging. There have been substantial global improvements in WASH access in the past two decades, but progress within Sub-Saharan Africa has lagged other regions [2]. Within Sub-Saharan Africa, rural areas have lower rates of access to WASH infrastructure and worse health outcomes than their urban counterparts [2–4]. For example, the Joint Monitoring Program reports that only 6% of the urban population in Sub-Saharan Africa uses unimproved sources of drinking water compared to over 25% of the rural population [5].

Geographically isolated communities and those with lower population density are more expensive to reach with behavior change messaging to improve safe WASH practices [6]. To transmit information to a large number of rural households, organizations may conduct mass media outreach via radio or television or repeated in-person community-based events. Alternatively, schools can be an attractive entry point, especially for child-focused programs, because they can engage students from many neighboring villages at once [7]. Most school-based WASH programs are designed to increase student knowledge and practice of behaviors such as washing hands with soap; some also include school WASH infrastructure improvements [8]. There has been substantial research into the impact of school-based WASH programs on improving water access [9–13], sanitation [14] and handwashing infrastructure or behavior [15–23], but almost entirely limited to changes in the school setting.

Architects of many school-based programs also envision that students will carry knowledge and behavior change messaging they learn at school to share with family members at home [10, 24–29]. Students in these programs are characterized as “agents of change.” It is important to note that increases in knowledge are necessary, but insufficient to lead to durable changes in caretaker behavior. Instead, we posit that using students as messengers of information acts to remind or encourage caregivers to practice a behavior that they may already know, but do not regularly perform. This in turn must occur in an enabling environment that supports the desired behavioral changes.

Research conducted in higher-income contexts has tested the viability of using school-aged children to encourage preventative health activities for parents, such as cancer screenings or physical exercise [30–32]. These studies have shown some promise and suggest a role for children to share information and remind parents to perform healthy behaviors. However, there are relatively few evaluations measuring the willingness and ability of children to transmit WASH-related messages from the school to the home in low-income countries and recent evaluations have measured little to no impact on caregiver behavior change [10, 24–29].

These disappointing outcomes are perhaps less surprising when considering the constellation of circumstances that need to exist for a WASH message delivered at school to be transmitted successfully by a student to family members at home. In order to improve the possibility of household behavior change, we believe that increased focus should be placed on understanding the “upstream” message sharing between students and their caregivers. Understanding where this exchange breaks down between the school and the home will lead to improved program design and outcomes. To the best of our knowledge, this question has not been addressed in prior evaluations of students’ capacity to act as agents of change in WASH programming.

In this study, we first present a conceptual model describing the conditions we hypothesize are necessary for messages from a school-based WASH curriculum to be relayed from students to their caregivers. Next, we operationalize this model using field data from Zambia to measure how a unique, school-based WASH program with a focus on students acting as agents of change affects the frequency of information transfer between schools and homes.

### Conceptual model

**Fig. 1:**
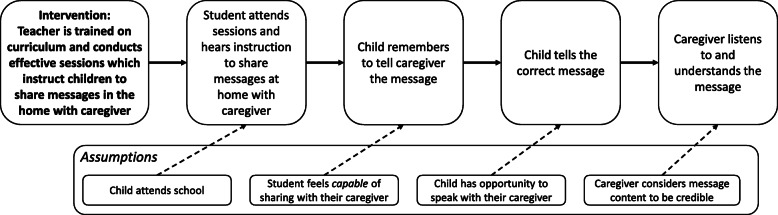
Simplified conceptual model of how students transmit messages from school to their caregiver at home in school-based programs with agent of change objectives

Our conceptual model (Fig. 1) was developed through a literature review and analysis of pilot data collected in Zambia during 2016. The model elaborates the hypothesized conditions necessary for successful transmission of information by students participating in a school-based educational program to their caregivers at home. Descriptions of the indicators used for each model element are included in Table S1 of the SI.

For students to share a message they learned in school with their caregivers successfully, they need to attend the session where the information is presented; be instructed to share the information; remember to share the message; have the opportunity to deliver it to a caregiver; and convey the information correctly. Several assumptions underpin the model as well. The student must attend school, have the opportunity to interact with the caregiver, and feel capable of and allowed to impart information to the caregiver without being asked. For the caregiver to listen to and understand the message, they must consider both the school and their child to be credible sources of information.

## Methods

### Intervention

Beginning in 2015, the international non-governmental organizations (NGOs) World Vision and Sesame Workshop collaboratively developed the WASH UP! program to improve school WASH infrastructure and increase knowledge and practice of safe WASH behaviors. WASH UP! combines a 12-week educational curriculum created by Sesame Workshop targeting students in grades 1–4 with school WASH infrastructure improvements carried out by World Vision. Program designers targeted these children in order to influence their behaviors during primary school, while they are hypothesized to still be developing habits. Teacher training is conducted by Sesame Workshop over the course of 3 days, and materials distribution and infrastructure improvements are funded and implemented by World Vision.

The curriculum includes 12 sessions, each of which is approximately 30–40 min long and uses child-friendly pedagogical strategies such as play-based learning. Activities include listening to the teacher read from a story book, playing interactive games, structured discussion of key curriculum messages, and watching two 10-min videos (see a complete list of activities in Table S2). All necessary materials, including play mats, board games, and projectors are provided at no cost to the schools. The twelve sessions are designed to be conducted before, during, or after normal school hours, at the discretion of the teacher. The key messages from the curriculum include admonitions to wash your hands with soap, use the latrine all the time, drink water from improved water sources, treat your drinking water, and to act as “agents of change.” Key curricular messages or homework assignments intended to help students act as an agent of change appeared five separate times in the 12-session program (Table 1).

**Table 1:** Activities and assignments in the WASH UP! curriculum supporting the learning objective of children acting as agents of change^a^

Session	Key Messages	Supporting Activities
1	• Introduce Raya and Elmo, the characters in the storybook • Introduce all learning objectives	• Teacher reads story of Elmo and Raya out loud to the class • Students are asked to teach someone at home one of the messages about healthy behaviors from the story
5	• Practice the healthy behaviors discussed in WASH UP! • Everyone is a teacher	• Students are asked to teach peers about healthy behaviors learned during WASH UP! during the session • Students are asked to teach a family member one of the healthy behaviors learned during WASH UP! after school
6	• Always wash your hands with soap after using the latrine • Washing your hands with soap will remove germs • Scrub between your fingers, on the front of the hand, and the back of the hand	• Students are asked to teach someone at home the handwashing song taught in class • Learning object was sent home with students to share with their caregivers
11	• Review ways to keep your school and home clean	• Students are told to tell someone at home about their “healthy superstar adventures”
12	• Review the different places, people, and activities that contribute to their health • Review the role of students in making sure the school environment is healthy and safe	• Students promise to continue taking care of their school toilets, water source, and handwashing stations • Students promise to continue teaching their friends about how to stay healthy and safe

^a^ A full list of activities, assignments, and learning objectives in the curriculum is provided in SI, Tables S2 and S3

### Study site, sample frame, and design

This project took advantage of the scheduled rollout of the WASH UP! program to the study district in southern Zambia during 2017–2018. Within this rural district, World Vision works primarily in 14 schools. Of these, two were excluded from the sample frame, one due to its distance from other schools and the other because it was a joint primary and secondary school whereas the others were exclusively primary schools.

All twelve study schools already had a functional, improved water point, and at least two gender-segregated, improved pit latrines prior to baseline data collection. The only impact on WASH infrastructure due to the WASH UP! program was the verbal encouragement by World Vision employees for schools to construct improvised handwashing stations, or “tippy taps” if they did not already have free standing handwashing stations. This verbal encouragement occurred in nine of the 12 schools.

All 12 schools in our sample frame were coeducational and served students from grades 1 to 7. Schools had an average enrollment of 589 (SD = 235) students. Several days prior to the day of data collection, we sent out invitations asking caregivers who had children in grades 1 or 4 to come to the school for interviews with the research team. All caregivers who came to the school were invited for interviews to discuss their child’s education, WASH practices, and knowledge (*N* = 729). At the beginning of the interview, the caregiver was informed that the school would be conducting a supplemental educational program and requested permission for their children to attend and be interviewed as part of a research project with a United States university. Caretakers were provided with a form to fill out with their child’s name and grade, and could provide consent for some, all, or none of their children to participate. Caregivers who were illiterate were read the form aloud, and a proxy signature of a friend or neighbor was used to certify their comprehension. Three declined to participate (< 1%). During endline data collection, caregivers were invited back using the same procedure. In 5% of cases, (*N* = 22) a different caregiver, usually a grandparent or spouse, was interviewed.

To capture the impact of the WASH UP! curriculum on students of different ages, we interviewed students in grades 1 and 4 (*N* = 761). By capturing students in grades 1 and 4, we were able to compare the endpoints of Sesame Workshop’s target population with each other to better isolate the role of age in study outcomes. All students whose caregiver consented for them to be interviewed were included in our sample frame. Assent was sought from the child prior to each interview. Five students (1%) declined to participate. We interviewed one teacher each from grade 1 and grade 4 in the 12 schools about program satisfaction, any challenges with implementation and potential future improvements (*N* = 24). These teachers reported being responsible for teaching the WASH UP! curriculum. All interviews of students, caregivers, and teachers consisted of structured surveys with a mix of open- and close-ended questions.

We used a quasi-experimental, prospective cohort study design. Due to ethical concerns raised by World Vision, we did not include schools that would not be receiving the WASH UP! curriculum in the sample frame. The nature of the interviews disrupted school sessions for students and teachers and required caregivers to come to the school for interviews. These efforts, in the absence of a clear intervention, were seen as a potential risk to the community relationships that World Vision had cultivated over several years. Therefore, all 12 schools where interviews were conducted received the combined intervention of WASH infrastructure support, teacher training and educational curriculum materials prior to endline data collection.

In addition to the standard WASH UP! curriculum, the research team conducted an experiment testing the hypothesis that providing a take-home object could support the objective of students acting as change agents. Within our causal model, this “learning object” reminds students of a specific message they have been asked to share with caregivers; it may also make this conversation more memorable for the caregiver. The learning object was a simple color printout of one image from the WASH UP! story book.

The learning object was distributed to a random subset of five of the 12 schools. Teachers provided the learning object to students with the instruction to take it home and discuss a key message about handwashing from the WASH UP! curriculum with their caregivers.

We report our methods and results in accordance with Transparent Reporting of Evaluations with Nonrandomized Designs (TREND) guidelines, with full checklist reported in the SI, Table S8 [33].

### Data collection

Data collection occurred in three periods: June 2017, September 2017, and June 2018. However, in early 2018, there was a cholera outbreak in Lusaka with minor spread to other provinces, including one case in a town near the study site. As a result, many of the public-health messages included in the WASH UP! curriculum, such as handwashing, water treatment, and latrine usage, were also being communicated by the government and mass media outlets. Due to the widespread distribution of these messages and lack of a control group of schools, we could not make claims about the differential impact of WASH UP! versus cholera-related messaging for the final round of data. Therefore, all data presented are from the first two rounds of data collection (i.e., before the cholera outbreak), which are referred to as baseline and endline, respectively.

During each round of data collection, the same schools were visited for interviews with students, caregivers, and teachers. Our research questions investigated the transmission of information from students to their caregivers at home. Therefore, students and caregivers were included in the data analysis only if both the student and their caregiver were interviewed during the same phase of data collection. All student and caregiver interviews were conducted separately. All efforts were made to interview the same respondents during both rounds of data collection. In instances where the caregiver had children in both grade 1 and grade 4, a random draw was performed by the enumerator prior to the interview to pair them with one of their children for the purpose of answering survey questions pertaining to their interactions with that specific child.

All interviews were conducted at the school grounds by twenty Zambian research assistants who had completed 2 weeks of intensive training and pretesting. The data-collection instruments were initially written in English, then translated into Tonga, the most commonly used local language of the region, by an external translator. All data were collected on tablet computers using SurveyCTO software.

The primary outcomes measured were student knowledge of key messages from the curriculum and self-reported frequency of children sharing WASH-related messages from school with their caregiver. The secondary outcomes were the self-reported frequency of caregivers reporting that their child shared a WASH-related message with them, self-reported changes in household infrastructure and caregiver behavior, and perceptions of the program by teachers and students.

Changes in knowledge were evaluated with survey questions related to key curricular messages. For example, students were shown pictures of different water sources in sequence. They were asked to assess if the source was safe or unsafe to drink water from. If they answered that the water was unsafe to drink, they were asked why. Enumerators were instructed to not prompt the respondent, only to encourage them to answer to the best of their knowledge. Each respondent was permitted to provide as many answers as s/he wished (see the full question text and correct responses in SI, Table S4).

The frequency with which students transmitted curricular messages was also measured through survey questions. Students were asked, *“During the previous school term, do you remember a teacher ever instructing you to share something you learned at school with your family?”*. Students who answered “yes” were asked to describe the subject matter of messages they shared. Similarly, caregivers were asked “*As far as you remember, did [your child] come to you to share a lesson(s) s/he learned at school at any time in the previous school term?*”. Those who answered yes were asked to describe the subject matter of messages that were shared. Messages that specifically pertained to water, sanitation, or hygiene were categorized as “WASH-related messages”. Those regarding curricular subjects or administrative fees were categorized as “Non-WASH-related messages”.

We collected data on the practice of WASH behaviors and the availability of WASH infrastructure at the home through self-reported answers to survey questions. The behaviors we measured were handwashing frequency, where adults in the household typically defecated and the frequency and type of water treatment used for drinking water. We also collected self-reported information on the presence of a dedicated place to wash hands in the home and the primary water source used for drinking water.

### Data analysis

**Fig. 2:**
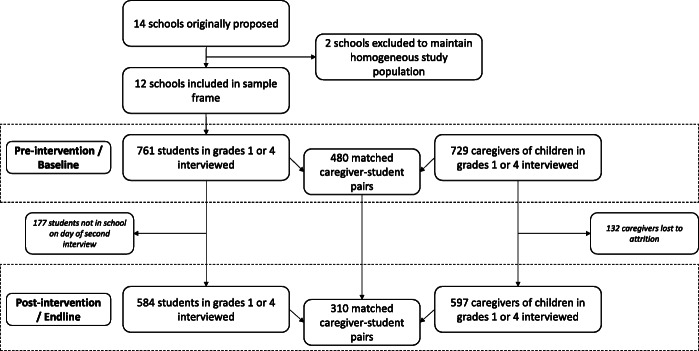
Flow chart of student and caregiver interview count. Matched caregiver-student pairs are instances in which the student and their caregiver were both interviewed in the same phase

Data were cleaned and analyzed using R version 3.6.1 (R Foundation, Vienna, Austria). Our research questions required elements of both the student and caregiver interviews to be included in our analyses. Therefore, we restricted our data analyses to include only responses where we interviewed both the student and their caregiver during the same data collection phase. This reduced our sample size from 761 students and 729 caregivers to 480 matched caregiver-student pairs at baseline. The reduction from 761 students and 729 caregivers to 480 matched caregiver-student pairs was driven by two factors. First, if a caregiver had multiple children in grade 1 and/or 4 they answered survey questions focusing on only one of their children. However, they typically gave consent for all their children to be interviewed. Therefore, for a single caregiver, we would have potentially interviewed multiple children. Second, the caregiver may have come to be interviewed on a day when the child did not attend school. We did not collect detailed data on reasons for student absence, but we were informed by teachers that students of all ages miss school with some regularity due to work or household chore requirements. At endline, the same procedure reduced our sample size from 584 students and 597 caregivers to 310 matched caregiver-student pairs (Fig. 2). Of these 310 matched pairs, 308 were previously interviewed at baseline; 2 pairs were new interviewees at endline only. We used chi-squared tests to identify differences in demographic indicators and primary outcomes between respondents who were lost to attrition after one round of data collection and those who did not.

Tests of changes in child knowledge, caregiver knowledge, and caregiver behaviors were evaluated over time using generalized linear mixed effects models, with random intercepts for each caregiver-child pair and for each teacher to account for household and classroom-level clustering, respectively. Changes in the odds of children reporting that they shared messages with their caregivers were measured using generalized linear mixed effects models with a log link with the same random intercepts as above, while also controlling for the grade level and sex of the student, whether the sex of the caregiver interviewed at the school matched the sex of their child who was interviewed, whether they attended a school that received the learning object, and an interaction term between receiving the learning object and time.

Two additional control variables were used to account for caregiver-child communication dynamics: 1) whether a child answered “yes” to the question of whether they “*could teach a caregiver something that you know that they don’t know,*” and 2) whether a caregiver reported that they “agreed completely” with the statement, “*It is important for my children to share what they learn in school with their caregivers at home.*” The same analysis method was used to calculate changes in the frequency of caregivers reporting that their child shared a message with them. We ensured that our mixed effects models met all assumptions for homogeneity, normality, and independence of residuals using the DHARMa package in R [34]. Odds ratios were converted to risk ratios due to high baseline prevalence of some outcomes using standard methods [35].

## Results

All twelve schools had an improved water source and at least two gender-segregated, improved latrines on-premises (Table 2). On the day of baseline data collection, 83% (*N* = 10) of schools had water available for handwashing and 42% (*N* = 5) had both water and soap available.

**Table 2:** Baseline characteristics of study schools (*N* = 12)

School characteristic	Value
Mean (SD) pupil to latrine ratio on the day of visit	75.0 (37.9)
Percentage of schools with functional handwashing stations with water on the day of visit	83%
Percentage of schools with functional handwashing stations with water and soap on the day of visit	42%
Proportion of schools with an improved water source on premises on the day of visit	100%
Proportion of schools with at least two gender-segregated, improved latrines on premises on the day of visit	100%
Total student enrollment per school (SD)	588 (226)

Of the 480 students included in the analysis at baseline, 53% were enrolled in grade 1 and the balance enrolled in grade 4 (Table 3). Student respondents were 51% female overall (48% of grade 1 students and 54% of grade 4 students). Students in grade 1 were 7.9 years old on average (SD = 1.8) and students in grade 4 were 11.2 years old on average (SD = 1.4).

**Table 3:** Baseline characteristics of sample population, by grade

Questions	Total (***N*** = 480)	Grade 1 (***N*** = 254)	Grade 4 (***N*** = 226)	Significance level of comparisons between grades
Percent of interviewed students who were female	51%	48%	54%	*p* = 0.26
Mean (SD) age of students	9.7 (2.3)	7.9 (1.8)	11.2 (1.4)	*p* < 0.001
Number of caregivers interviewed	480	254	226	*p* = 0.08
% female caregivers interviewed	78%	82%	74%	*p* = 0.05
Mean (SD) age of caregivers	38.0 (10.1)	35.8 (9.7)	40.4 (10.0)	*p* < 0.001

Tests of significance conducted between grade 1 and grade 4 respondents with student t-tests for continuous variables and chi-squared tests for binary variables

A large majority of caregivers of the 480 students were female (78% overall) (Table 3). Caregivers were an average of 38.0 years old (SD = 10.1).

### Perceptions of WASH UP!

Students and teachers overwhelmingly reported the curriculum to be enjoyable and engaging. Students across both grades identified it as ‘fun’ (97%) and ‘interesting’ (98%). Teachers had similarly positive reviews of the program; 96% of them found the content ‘important’ and 88% reported that they planned to teach it in the school term following the end of the study. Teachers reported that the information in the curriculum was not entirely new, however, especially for students in grade 4. We found that 23% of grade 1 teachers reported that the information presented in WASH UP! was entirely new to their students versus just 7% of grade 4 teachers.

Teachers reported conducting an average of 6.3 (SD = 2.8) WASH UP! sessions during the 13-week academic term. When asked if there were particular reasons they did not complete the entire 12-session curriculum, most teachers reported that they had preexisting time commitments. For example, 13 of 24 teachers (54%) reported that required supervision of other school clubs was a key factor in limiting their ability to conduct WASH UP! sessions as often as they would have liked. In addition, projectors and videos were included as part of the curricular materials, but three of the twelve schools did not have electricity, making one of the twelve sessions much more challenging to administer.

### Changes in knowledge

Changes in student knowledge of key messages from the curriculum were particularly pronounced among grade 1 students (Table 4). Students in grade 1 were significantly more likely after the intervention to be able to describe germs in a scientifically valid way, identify images of “unsafe” sources of drinking water and identify contamination risks in river water. The recognition of taps and boreholes as “safe” sources of drinking water was consistently high (72–79%) across time periods for both grade levels. At baseline, grade 4 students had significantly higher knowledge of all key messages than grade 1 students. Among grade 4 students, there were no significant changes in knowledge of germs or safe water source identification. There were improvements, although smaller than among grade 1 students, in the proportion who were able to correctly identify unsafe water sources and potential contamination in river water. Full details of how knowledge measurements were calculated are shown in Table S4.

**Table 4:** Knowledge of key messages among students by grade and study phase

	Grade 1			Grade 4		
	*Baseline*	*Endline*	*p-value*	*Baseline*	*Endline*	*p-value*
% who were able to accurately state what germs are	24%	37%	***p*** **= 0.008**	51%	56%	*p* = 0.27
% identifying “safe” sources of drinking water	79%	74%	*P* = 0.14	72%	75%	*p* = 0.49
% identifying “unsafe” sources of drinking water	46%	64%	**p < 0.001**	84%	91%	***p*** **= 0.03**
% correctly identifying potential contamination in river	22%	54%	**p < 0.001**	74%	86%	***p*** **= 0.005**
Number of observations	254	152		226	158	

Significance levels for endline vs. baseline within a grade, calculated from generalized linear mixed effects models, allowing for random intercepts at the individual respondent and school levels

We measured no significant changes in caregiver knowledge of key messages taught in the WASH UP! curriculum. At baseline, knowledge of all key messages for caregivers was already above 80%, indicating high levels of awareness prior to the intervention (Table S5).

### Changes in self-reported message transmission

**Fig. 3:**
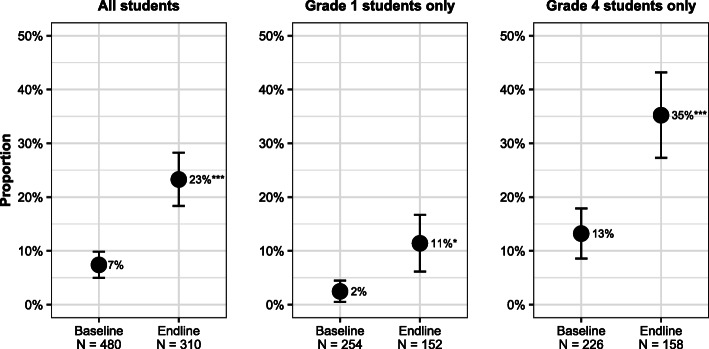
Proportion of students who reported sharing a WASH-related message with their caregiver in the previous term, by grade level and study phase. Significant difference between baseline and endline: ^*^ 0.01 ≤ *p* < 0.05, ^***^ *p* < 0.001

At baseline, 7% of all students reported sharing a WASH message at home with their caregivers during the previous school term. This increased significantly (*p* < 0.001) to 23% after program exposure, at endline (Fig. 3). Baseline values and the magnitude of change over time both differed by grade. At baseline, 2% of students in grade 1 reported sharing a WASH message with their caregivers; this rose to 11% at endline (*p* = 0.01). Among grade 4 students, the share rose from 13 to 35% (*p* < 0.001).

**Fig. 4:**
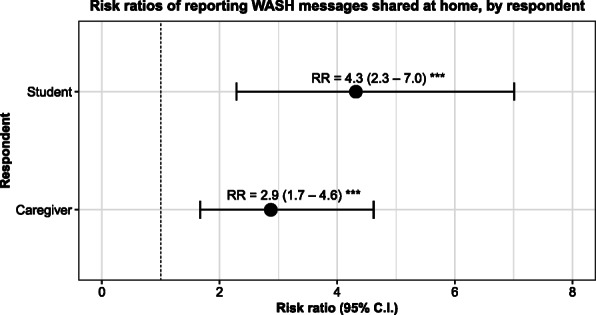
Risk ratios and 95% confidence interval of students and caregivers reporting WASH messages were shared at home associated with program exposure. Total of 790 observations from 480 unique student-caregiver pairs. Significant difference between baseline and endline: ^***^ *p* < 0.001. Full model outputs are included shown in Tables 5 (student) and S7 (caregiver)

Students reported sharing WASH messages 4.3 times more frequently after the intervention (Adjusted Risk Ratio, ARR = 4.3, 95% C.I. = (2.3–7.0); *p* < 0.001) (Fig. 4). We measured no significant changes in the frequency with which students reported sharing of non-WASH messages, such as paying school fees, math, or other curricular subjects (ARR = 1.5, 95% C. I = (0.9–2.1); *p* = 0.08).

Among caregivers, a similar pattern was observed. Caregivers reported that their children shared WASH messages with them 2.9 times more frequently (ARR = 2.9, 95% C.I. = (1.7–4.6); p < 0.001) but reported no significant change in the frequency with which they shared non-WASH messages (ARR = 1.0, 95% C.I. = (0.8–1.1); *p* = 0.75).

### Factors associated with message transmission

Students in grade 4 at the start of the program were 5.2 times more likely to report sharing a WASH-related message with their caregiver(s) (ARR = 5.2, 95% C.I. = (2.3, 8.9); p < 0.001, reference = grade 1) (Table 5). There were no significant associations between sex of the student interviewed or receipt of the learning object and reporting having shared a WASH message with their caregiver. Similarly, we found no significant association between changes in message sharing and instances where the sex of the student and the caregiver being interviewed matched.

**Table 5:** Output of generalized linear mixed effects model estimating the relative risk of a student reporting sharing a WASH-related message with their caregiver

*Predictors*	*Relative Risk (95% Confidence Interval)*	*P-value*
**Post-exposure (ref = baseline, pre-exposure)**	**4.3 (2.3, 7.0)**	**< 0.001**
**Grade 4 student (reference = Grade 1 student)**	**5.2 (2.3, 8.9)**	**< 0.001**
Sex = Male student (reference = Female student)	1.1 (0.5, 2.1)	0.85
Child reports being able to teach caregivers something they don’t know	1.2 (0.5, 2.5)	0.69
Caregivers “completely agree” that their children should share what they learn	2.8 (0.9, 6.5)	0.07
Sex match (ref = sex is the same between caregiver and student)	1.8 (0.9, 3.0)	0.05
Learning object subgroup assignment (ref = not assigned to subgroup analysis)	1.9 (0.6, 5.0)	0.25
Learning object x Exposure	1.3 (0.4, 3.5)	0.65
Total Observations		790
Conditional R^2^		0.59

* 0.01 ≤ *p* < 0.05 ** 0.001 ≤ *p* < 0.01 *** *p* < 0.001

To measure intrafamily dynamics that could affect child behavior, we asked questions regarding child-caregiver contact. At baseline, 77% of children answered “yes” to the question of whether they “could teach a caregiver something that you know that they don’t know” and 87% of caregivers reported that they agreed completely with the statement, “It is important for my children to share what they learn in school with their caregivers at home.” These values significantly increased to 87% (*p* < 0.001) and 94% (*p* = 0.01), respectively, at endline. Neither of these variables were significantly associated with the primary outcome in the multivariate model (Table 5).

### Changes in self-reported household WASH infrastructure and behaviors

We measured no significant changes in household WASH infrastructure or self-reported WASH behaviors. At baseline, 47% of respondents reported having a dedicated place to wash their hands. This rose to 56% at endline, but this change did not represent a statistically significant difference (Table S6). The share of households reporting the use of a shared or private latrine for defecation also did not significantly change after the intervention (59 to 56%). The proportion of households reporting the use of an improved water source as their primary source of drinking water was 75% at baseline and 76% at endline. Similarly, the proportion of households which reported using some type of water treatment (either filtration or chlorination) “approximately half the time” or “always or most of the time” did not significantly change (18 to 20%).

### Attrition analysis

We measured relatively high levels of attrition, as 480 caregiver-student pairs were interviewed at baseline and only 310 of those pairs (65%) were able to be interviewed at endline. Among caregivers, we investigated whether there were significant differences between respondents who were lost to attrition and those who were not across the following self-reported variables: use of an improved water source, presence of a dedicated place to wash hands at home, use of a shared or private latrine for adults to defecate, trust of schools and frequency of speaking to their children about school. We measured no significant differences (Table S9).

We conducted a similar analysis among students, measuring differences across curricular knowledge, reported sharing of WASH messages with their caregivers, and reported comfort teaching their caregivers something new. Students who were lost to attrition were significantly less likely to correctly identify surface water sources and shallow wells as unsafe sources of drinking water (53% versus 70%, *Χ*^2^ = 13.7, *p* < 0.001). We measured no other significant differences (Table S10).

## Discussion

The WASH UP! program, a high-intensity, school-based WASH intervention, was associated with significant improvements of student knowledge of key messages. Students exposed to the program also reported significantly higher rates of spreading messages from school to the home. The substantial differences in these outcomes between grades suggest that school-based WASH programs may benefit from targeting students of different grades with more age-appropriate programming.

We measured large, significant increases in knowledge of the key messages among students in grade 1. However, we measured more modest increases in knowledge among grade 4 students, due in part to the fact that they had comparatively high levels of baseline knowledge. This is corroborated by findings from our teacher surveys, where more teachers in grade 1 reported that the information presented in the curriculum was new to their students compared with teachers in grade 4 (23% vs 7%). While it is possible that students would discuss amongst themselves the content of the interview, this potential limitation is minimized by the fact that school was actively in session during our interviews. Therefore, students were re-entering an active classroom after the interview, which we believe would reduce the possibility of extended exchanges with classmates about perceptions of “correct” answers. In contrast, caregiver knowledge did not significantly increase over the study period. However, we note that our hypothesized conceptual model is not reliant on children teaching their caregivers new information. Rather, the novel act of a child reminding their caregiver to perform an action is the pathway of primary interest.

Students in grade 4 were 5-fold more likely to report sharing a WASH-related message with their caregivers than students in grade 1 after program exposure. This finding indicates that while older students may not have increased their knowledge of curricular messages as much as younger students, they were much more successful in carrying these messages home. Students in grade 4 may be more effective agents of change due to higher self-efficacy, aptitude at recalling what they were instructed to do by their teacher, and/or higher standing in the home social environment. Therefore, reinforcing these attributes in older students while spending less time on repetitive material may be a programmatic adjustment that could increase the communication of curricular messages from students to their caregivers. We acknowledge however, that the heterogeneous impact on younger versus older students could be driven in part by older students being more adept at anticipating a perceived “correct” answer during a survey. This type of response bias has been measured to be most pronounced in instances where the respondent has volunteered their participation and/or where the enumerator fails to keep a neutral stance in questioning [36, 37]. In our study, students were volunteered by their caregivers, and enumerators were specifically trained during their intensive 2-week training led by the authors on strategies for neutral questioning. We believe that these factors limit the potential impact of this type of response bias on our findings.

Students in both grades reported feeling comfortable sharing messages they learned in school with their caregivers. Likewise, caregivers reported having an interest in their child’s schoolwork and speaking to them frequently about what they are learning. These features were hypothesized in our conceptual model to be necessary conditions for students to act as agents of change. While these responses are self-reported, they align with previous work in Zambia, Kenya, and Tanzania that reports similar levels of openness among adults to their children regarding health-related information [24, 25, 38]. However, we did not intervene in the community or with caregivers, and thus may have missed opportunities to further increase student-caregiver exchange.

We found that introducing a take-home “learning object” in five of the 12 schools was not significantly associated with any changes in the frequency of sharing WASH messages at home (Table 5). Based on findings from a previous study in Kenya [29], we hypothesized that being given a colorful printout with a pictorial representation of a key message would address three points on our conceptual model. Specifically, we believed that receiving the object would remind students to tell their caregiver the message, refresh their memory on the correct message to tell, and make the event more memorable to their caregiver. None of these three hypotheses were supported by our survey data. It is possible that the handouts were not distributed at the correct time or with the correct instructions or were perceived as banal and did not prompt student-caregiver interaction. We believe that it is most likely that a single extra handout did not represent a substantial marginal encouragement over the WASH UP! curriculum as a whole. We believe that future implementations of a similar concept should focus on more durable items that directly encourage students to start conversations and interact with their caregivers. Related research has used calendars as a mode of accountability for handwashing at home [39], which could be repurposed as a learning object to also transfer information to the home.

Teachers reported having other obligations that precluded them from conducting all the WASH UP! sessions within a single academic term. Due to preexisting after-school programs, clubs, and sports, some teachers were unable to maintain a weekly schedule for WASH UP! sessions for all grades. Although every teacher reported covering all curricular messages at least once, there is evidence from the child education literature that there are benefits to comprehension with message repetition [40]. If, for example, a teacher conducted six out of the 12 sessions, they would repeat the agent of change messaging only three out of the intended five times, potentially reducing impact.

We measured no changes in self-reported household WASH infrastructure or behaviors, specifically the use of a shared or private latrine, presence of a dedicated place for handwashing, or point-of-use water treatment. Due to operational limitations, we were unable to directly measure these indicators at the caregivers’ homes through visual inspection or water testing. Therefore, while these indicators did not significantly change over time, we believe that these findings should be interpreted with caution due to the collection of data on school grounds. Conducting the interviews at schools also introduced potential selection bias in which caregivers participated. We did not target any specific caregivers for inclusion, but by holding interviews at school, we interviewed caregivers who were willing and able to walk to school during the workday for an interview offering no compensation. These caregivers may potentially be more involved in their child’s education and may discuss their schoolwork more frequently at home. This suggests that our findings of limited message transmission to the home may represent an optimistic case relative to a random sample of caregivers.

In addition, by conducting a before-and-after evaluation with no control group, there is a risk of assigning impact to the WASH UP! intervention that may be alternatively explained by broader changes in the region. While we measured large increases in knowledge of key messages among grade 1 students, it is possible that this may have been due solely to their attendance at schools rather than the WASH UP! program. However, the rapid evaluation timeline – measuring outcomes immediately before and after the 3-month program – mitigates some of this concern. It is possible that younger students would dramatically increase their knowledge of key WASH messages over that particular 3-month period in the absence of the WASH UP! intervention, but this was not reflected in our findings from teacher interviews. Similarly, there is no evidence of educational policy shifts that would drive changes in the reported frequency of students reporting to share messages with their caregivers. We did not ask about the existence of previous WASH programs in the schools or nearby village in our interviews with caregivers, teachers, or students. Therefore, if these programs occurred in the recent past, our findings may represent the combined impact of the WASH UP! program with other similar programming.

Finally, due to our use of merged caregiver and child datasets, if either the caregiver or the student was not able to be interviewed, the paired respondent was considered to have been lost to attrition. This resulted in relatively high levels of attrition. In our attrition analysis, we found that students who were only interviewed once had significantly lower baseline knowledge of unsafe sources of drinking water, but no other significant differences from students interviewed twice. Therefore, while student attrition may bias our estimates of the associations between program exposure and knowledge of unsafe water sources upward, we found no evidence that attrition biased other reported findings.

The findings of this study emphasize that child-focused, school-based programming must consider differences in student ages and capabilities in their design and implementation. Overall, we find that while our hypothesized preconditions for students to act as agents of change were met, we observed only modest increases in self-reported message transmission from the school to the home. This suggests that relying exclusively on young children, in the absence of more direct interventions in the community and/or home, is an unrealistic strategy to achieve community-level knowledge improvements. However, this overall finding masks the divergent outcomes we measured for students in grade 1 versus grade 4. Targeting students in grade 1 was associated with important knowledge benefits, but these, at least in the short term, only accrue to the child. However, students in grade 4 were significantly more likely to report transmitting messages from the school to the home, potentially providing more community-level value. Programmatic adjustments that focus time and resources on knowledge acquisition for younger students and empowering older students to act as agents of change may provide a larger benefit to students and caregivers alike. Adding activities such as peer mentoring to the curriculum would integrate constructs from social cognitive theory (eg mastery learning or reinforcement) [41] with the existing program, potentially increasing its impact.

## Supplementary Information

**Additional file 1.**

## Data Availability

The data used in this study are a part of the doctoral dissertation of the first author and are not publicly available. Anonymized data, survey instruments (in English), and analysis code are available from the corresponding author upon reasonable request and signature of a mutual agreement.
