# Supplementary material for: The potential of school-based WASH programming to support children as agents of change in rural Zambian households
Source: BMC Public Health. 2021 Oct 8;21:1812. doi: 10.1186/s12889-021-11824-3 (PMC8501527; doi:10.1186/s12889-021-11824-3)
Supplement: Supplementary file 1 — Additional file 1. [file 12889_2021_11824_MOESM1_ESM.docx]

# Supplementary Information

## Table S1. Constructs and indicators for each level of the proposed conceptual model

| **Actor** | **Construct** | **Indicator** |
| --- | --- | --- |
| Teacher | Teacher understands the key messages of the WASH UP! curriculum | Teacher attended the training workshop conducted by Sesame Workshop prior to the launch of the program in schools |
| Student | Student attendance at school | Average attendance by grade |
|  | Student hear this call to action | Student responds “Yes” when asked whether they remember being told by their teacher to share a message from school with their parent  Question text: During the previous school term, do you remember a teacher ever instructing you to share something you learned at school with your family? [Yes, No, Don’t know] |
|  | Student feels capable of sharing [information] with their parent | Student responds “Yes, I’m sure” when asked whether they feel capable of sharing new information with their parent  Question text: Do you think you could teach a parent something that you know and they don't know? [Yes, I’m sure; No, I’m not sure; Don’t know] |
|  | Student has opportunity to speak with their parent  [about what they learned in school] | Parent reports that they speak to their child “once a day or more” about what they learned in school  Question text: “Approximately how often do you converse with your child about WHAT THEY LEARNED IN SCHOOL?” [Once per day or more, 2-4 times per week, once per week, every other week, less than every other week] |
|  | Student shares a message from the WASH UP! curriculum with their parent | Student responds yes to whether they shared a message in the previous school term with their parent AND specifies that it was a message related to water, sanitation, hygiene, health, or the WASH UP! curriculum  Question text: Were you able to talk to your family about those topics [your teacher asked you to share]? |
| Parent | Parent trusts the information their student learns in school | Parent strongly agrees that the information their child learns in school is trustworthy  Question text: I trust the information that my child tells me they learned in school” [strongly agree, somewhat agree, somewhat disagree, strongly disagree] |
|  | Parent remembers their child telling them a message from the WASH UP! curriculum recently | Parent responds that they heard their child share a WASH-specific message with them during the most recent school term  Question text: As far as you remember, did your student come to you to share a lesson(s) s/he learned at school at any time in the previous school term? [Yes, no, don’t know]  If yes: What topics did your student come to you to share during the previous school term? [water, sanitation, hygiene, health, WASH UP! specifically, other school-related messages] |

## Table S2. Full List of activities and assignments associated with WASH UP! curriculum

| **Session** | **Key Messages** | **Supporting Activities** |
| --- | --- | --- |
| 1 | - Introduce Raya and Elmo, the characters in the storybook - Introduce all key messages for the first time | - Teacher reads story of Elmo and Raya out loud to the class - Students asked to teach someone at home one of the messages about healthy behaviors from the story |
| 2 | - Germs are invisible - Germs can make you sick | - Students asked to identify places where germs can be found in the school environment - Students play “germ tag”, a game where touching someone spreads infection to that person |
| 3 | - Water from a borehole is safe - Water can look clean but still have germs - Boiling and chlorine can kill germs | - Students play a game where they learn to associate water taps and boreholes with providing safer drinking water (that still may need to be purified) - Students asked to count the number of boreholes between the school and their home |
| 4 | - Keep the school latrine clean - Always use the latrine to pee and poop | - Students asked to identify what items they need to bring with them to the latrine to use it safely - Students asked to inspect the school latrines and identify which are clean and which are dirty |
| 5 | - Practice the healthy behaviors discussed in WASH UP! - Everyone is a teacher | - Students asked to teach peers about healthy behaviors learned during WASH UP! during the session - Students asked to teach a family member one of the healthy learned during WASH UP! after school |
| 6 | - Always wash your hands with soap after using the latrine - Washing your hands with soap will remove germs - Scrub between your fingers, on the front of the hand, and the back of the hand | - Students asked to teach someone at home the handwashing song taught in class |
| 7 | - Carry water carefully so it does not spill - Do not waste water or play with it - Wash vegetables before cooking them - Wash your hands before preparing food | - Students asked to monitor the school water source and teach anyone who plays with the pump or is spilling water why it is important to care for the water source |
| 8 | - Throw all trash in a rubbish pit to keep the school environment clean - Always wear sandals into the latrine to avoid germs | - Students asked to come up with ideas for how to keep the school latrines clean |
| 9 | - Review handwashing messaging | - Students asked to come up with ideas for how to make sure that the school handwashing stations always have soap and water available |
| 10 | - Review water storage, treatment, and latrine messages | - Students watch short animated video featuring Raya and Elmo, reviewing key messages from WASH UP! |
| 11 | - Review ways to keep your school and home clean | - Students told to tell someone at home about their “healthy superstar adventures” |
| 12 | - Review the different places, people, and activities that contribute to their health - Review the role of students in making sure the school environment is healthy and safe | - Students promise to continue taking care of their school toilets, water source, and handwashing stations - Students promise to continue teaching their friends about how to stay healthy and safe |

## Table S3. Full list of learning objectives from the WASH UP! curriculum

| **Session** | **Topic** | **Learning Objective** |
| --- | --- | --- |
| 1 | Water | - Distinguish between safe and unsafe water sources - Choose to drink safe water - Transport, store, and handle drinking water in a safe way - Use water in a responsible way |
| 2 | Sanitation | - Use safe and inclusive latrines in a dignified way - Put rubbish in designated places - Understand the link between feces, rubbish, and bad health/disease |
| 3 | Hygiene | - Wash their hands properly and at appropriate times - Understand the importance of good personal hygiene - Practice good personal hygiene |
| 4 | Health Promotion | - Teach children and adults about water, sanitation and hygiene - Support health and hygiene at school, caring for water points, latrines, and hand washing stations |

## Table S4. Question text for measures of knowledge for students and caregivers

**Students:**

| **Row names in Table 3** | **Full question text (Instructions to enumerator in italics, question logic in brackets)** |
| --- | --- |
| % who were able to accurately state what germs are | Now I’d like to ask you about germs. Have you ever heard the word ‘germs’ before?  [If yes] What can you tell me about germs? What are they? (DO NOT PROMPT except ‘Anything else?’ Tick all responses mentioned by respondent.)  Credit awarded if student mentions that germs cause disease / make you sick and/or are invisible / are too small to see. |
| % identifying “safe” sources of drinking water | Now I’d like you to look at some pictures of different places where we can get water. For each one, can you tell me if you think the water we would collect would be safe for drinking?  Boreholes and taps are coded as safe. |
| % identifying “unsafe” sources of drinking water | Now I’d like you to look at some pictures of different places where we can get water. For each one, can you tell me if you think the water we would collect would be safe for drinking?  Uncovered well and surface water sources are coded as unsafe. |
| % correctly identifying potential contamination in river | [If river water identified as unsafe] What do you think is in the water from the river that makes it unsafe to drink? (DO NOT PROMPT except ‘Anything else?’ Tick all responses mentioned by respondent.)  Credit awarded if student mentions that sewage and/or germs and/or feces and/or human waste are sources of potential contamination. |

**Caregiver:**

| **Row names in Table 4** | **Full question text (Instructions to enumerator in italics, question logic in brackets)** |
| --- | --- |
| % identifying “before eating” as a critical time to wash hands (unprompted) | Can you tell me AT WHAT TIMES you think it is most important to wash your hands? (DO NOT PROMPT, other than “Any other reason?” Tick ALL answers mentioned by respondent.) |
| % identifying “after using the toilet” as a critical time to wash hands (unprompted) | Can you tell me AT WHAT TIMES you think it is most important to wash your hands? (DO NOT PROMPT, other than “Any other reason?” Tick ALL answers mentioned by respondent.) |
| % agreeing completely that “washing hands will prevent our family from getting diarrhea” | Please tell me if you AGREE COMPLETELY, AGREE SOMEWHAT, DISAGREE SOMEWHAT, or TOTALLY DISAGREE with it. Remember: there are no right or wrong answers. I really want to know what you think. Ready?  Washing hands will PREVENT our family from getting DIARRHEA |
| % disagreeing completely that “washing hands with water makes them just as clean as washing them with soap” | Please tell me if you AGREE COMPLETELY, AGREE SOMEWHAT, DISAGREE SOMEWHAT, or TOTALLY DISAGREE with it. Remember: there are no right or wrong answers. I really want to know what you think. Ready?  Washing hands with WATER makes them just as clean as washing with water AND SOAP. |

## Table S5. Knowledge of key messages among caregivers before and after WASH UP! program

|  | Baseline | Endline | p-value |
| --- | --- | --- | --- |
| % identifying “before eating” as a critical time to wash hands (unprompted) | 81% | 80% | n.s. |
| % identifying “after using the toilet” as a critical time to wash hands (unprompted) | 90% | 92% | n.s. |
| % agreeing “completely” that “washing hands will prevent our family from getting diarrhea” | 92% | 96% | n.s. |
| % disagreeing “completely” that “washing hands with water makes them just as clean as washing them with soap” | 83% | 79% | n.s. |
| Number of observations | 480 | 310 |  |

Significance levels calculated from generalized linear mixed effects models, controlling for gender of student, gender of caretaker that was interviewed, school, allowing for random intercepts at the individual respondent and school level
Significant difference between baseline and endline

## Table S6. Changes in self-reported behavior change among caregivers, by activity and study phase

| Questions | Pre-exposure | Post-exposure | p-value |
| --- | --- | --- | --- |
| % reporting having a specific place to wash hands after relieving themselves | 47%  (N = 348)^1^ | 56% (N = 310) | p = 0.2 |
| % reporting that adults usually use a shared or private latrine to defecate at home | 59%  (N = 480) | 56% (N = 310) | p = 0.8 |
| % reporting the use of an improved water source as their primary source of drinking water | 75%  (N = 480) | 76%  (N = 310) | p = 0.9 |
| % reporting treating household drinking water “about half the time” or more | 18% (N = 480) | 20% (N = 310) | p = 0.5 |

^1^This question was added to the survey on the third day of data collection, resulting in the collection of fewer responses
Significance levels calculated from generalized linear mixed effects models, controlling for gender of student, gender of caretaker that was interviewed, school, allowing for random intercepts at the individual respondent and school level

We measured no difference in self-reported behavior change among caregiver interviews in water treatment, use of a shared or private latrine for defecation, or having access to a specific place to wash hands after relieving themselves. There was a marginal increase in the percentage of caregivers reporting having a specific place to wash their hands after relieving themselves (47% to 56%), but this difference was not statistically significant.

## Table S7. Output of generalized linear mixed effects model estimating the relative risk of a caregiver reporting their child sharing a WASH-related message with them, allowing for random intercepts at the individual respondent and school level

| Predictors | Relative Risk (95% Confidence Interval) |  |
| --- | --- | --- |
| **Exposure to curriculum  (ref = baseline, pre-exposure)** | **2.0 *****  **(1.7, 4.6)** |  |
| Grade  (ref = grade 1) | 0.6  (0.3, 1.1) |  |
| Sex  (ref = female) | 0.8  (0.4, 1.6) |  |
| Child reports being able to teach parents something they don’t know | 0.9 (0.5, 1.6) |  |
| Caregivers “completely agree” that their children should share what they learn | 1.2 (0.5, 2.7) |  |
| Sex match  (ref = gender is the same between caregiver and student) | 1.1  (0.7, 1.7) |  |
| Learning object subgroup assignment  (ref = not assigned to subgroup analysis) | 1.7  (0.8, 3.4) |  |
| Learning object x Exposure | 0.6  (0.2, 1.5) |  |
| Total Observations | 716 | |
| Conditional R^2^ | 0.29 | |

* 0.01 ≤ p <0.05   ** 0.001 ≤ p < 0.01   *** p < 0.001

## Table S8. Transparent Reporting of Evaluations with Nonrandomized Designs (TREND) checklist

| **Paper Section** | **Item No** | **Descriptor** | **Inclusion?** |
| --- | --- | --- | --- |
| Title and Abstract | 1 | Information on how unit were allocated to interventions | This study is a non-randomized quasi-experimental study. Twelve schools were selected for participation based on where the NGO implementer already worked in the district. No control schools were included in the design. |
| Title and Abstract | 1 | Structured abstract recommended | Please see abstract |
| Title and Abstract | 1 | Information on target population or study sample | Study sample is 480 students and 480 caregivers from 12 schools in the Southern province of Zambia |
| Background | 2 | Scientific background and explanation of rationale | Conceptual model is included as Figure 1, laying out the scientific background and explanation of rationale. |
| Background | 2 | Theories used in designing behavioral interventions | The behavioral intervention was designed by Sesame Workshop, using their content development process. The research team did not play a role in this process. |
| Participants | 3 | Eligibility criteria for participants, including criteria at different levels in recruitment/sampling plan (e.g., cities, clinics, subjects) | Caregivers were eligible for participation if they had a child enrolled in grades 1 or 4 of one of the 12 schools at the time of baseline data collection. Caregivers were asked to come to the school on one of two days to provide consent for their child to participate. All students who we had permission to speak with were included int he sample frame. |
|  | 3 | Method of recruitment (e.g., referral, self-selection), including the sampling method if a systematic sampling plan was implemented | All caregivers who voluntarily came to the school grounds for an interview were included in the sample frame. If a parent had multiple children in grade 1 or 4, one of them was randomly selected using a random number draw. |
|  | 3 | Recruitment setting | The recruitment took place at the school either at a meeting that was scheduled by the implementing partner, World Vision, or during the day of baseline data collection, where parents were invited to come to the school for an interview. No compensation was offered. |
|  | 3 | Settings and locations where the data were collected | All data was collected from respondents on school grounds. |
| Interventions | 4 | Details of the interventions intended for each study condition and how and when they were actually administered, specifically including: | |
|  | 4 | Content: what was given? | The intervention was a 12-session curriculum on water, sanitation, and hygiene knowledge, attitudes, and perceptions. The curriculum used play-based learning strategies, storybooks, and interactive games as pedagogical strategies. |
|  | 4 | Delivery method: how was the content given? | The intervention was delivered during, before, or after school (at the discretion of the teacher). |
|  | 4 | Unit of delivery: how were the subjects grouped during delivery? | The unit of delivery was at the level of the school and the village(s) that the school drew students from. |
|  | 4 | Deliverer: who delivered the intervention? | World Vision, an international NGO with significant, long-standing local presence, delivered the intervention. They sourced materials and skilled labor for WASH infrastructure. Teacher training was provided by both Sesame Workshop and World Vision. All curricular materials were designed by Sesame Workshop with local input. |
|  | 4 | Setting: where was the intervention delivered? | The intervention was delivered in the 12 treatment schools |
|  | 4 | Exposure quantity and duration: how many sessions or episodes or events were intended to be delivered? How long were they intended to last? | The school curriculum was 12-sessions. Each session was designed to last approximately 30-45 minutes, and intended to be conducted once per week. |
|  | 4 | Time span: how long was it intended to take to deliver the intervention to each unit? | It was intended for the curriculum to be delivered over a 14 week time period. One week was devoted to training, 12 weeks for the curriculum, and one week for a mandatory national exam period. |
|  | 4 | Activities to increase compliance or adherence (e.g., incentives) | There were no incentives to participate in the curriculum activities or to be interviewed. Students who participated were given a token gift of a sticker or a pencil. |
| Objectives | 5 | Specific objectives and hypotheses | The objective was to understand how much a school-based WASH program would affect (1) knowledge, attitudes, and perceptions of the target students and (2) the frequency which students told their caregivers about what they learned in school. |
| Outcomes | 6 | Clearly defined primary and secondary outcome measures | Primary outcome 1: Percentage of students providing correct answers to survey questions designed to measure knowledge of key curricular messages.  Primary outcome 2: Percentage of students reporting sharing a WASH-related message with their caregivers at home.  Secondary outcome 1: Percentage of caregivers reporting their child sharing a WASH-related message with them at home. |
|  | 6 | Methods used to collect data and any methods used to enhance the quality of measurements | Surveys were conducted in the local language by Zambian enumerators to collect self-reported data. |
|  | 6 | Information on validated instruments such as psychometric and biometric properties | Surveys were translated into Tonga, the local language from English, and reviewed by enumerators fluent in both languages. |
| Samples size | 7 | How sample size was determined and, when applicable, explanation of any interim analyses and stopping rules | Sample size was determined in collaboration with the implementing partner based on budget and logistic considerations. This study was designed as a pilot study for a larger, scale-up trial and as a result, was not powered to detect outcomes. |
| Assignment method | 8 | Unit of assignment (the unit being assigned to study condition, e.g., individual, group, community) | The unit of assignment was a school. All students in a treatment school received the treatment. |
|  | 8 | Method used to assign units to study conditions, including details of any restriction (e.g., blocking, stratification, minimization) | The villages were selected by the implementing partner as part of their scheduled roll out of the WASH UP! curriculum across their catchment areas. Two schools were not included in the sample frame due to extenuating conditions (distance and having an attached boarding school). |
|  | 8 | Inclusion of aspects employed to help minimize potential bias induced due to non-randomization (e.g., matching) | Data was collected using panel methods such that students and caregivers were compared to each other across all outcomes. |
| Blinding | 9 | Whether or not participants, those administering the interventions, and those assessing the outcomes were blinded to study condition assignment; if so, statement regarding how the blinding was accomplished and how it was assessed. | No blinding was possible due the nature of the educational curriculum. |
| Unit of Analysis | 10 | Description of the smallest unit that is being analyzed to assess intervention effects (e.g., individual, group, or community) | The smallest unit of analysis is the individual (i.e. student and caregiver) level. |
|  | 10 | If the unit of analysis differs from the unit of assignment, the analytical method used to account for this (e.g., adjusting the standard error estimates by the design effect or using multilevel analysis) | We cluster at the child-caregiver pair level and the grade level-school in all analyses. This is accomplished using two different random intercepts in generalized linear mixed effects models. |
| Statistical Methods | 11 | Statistical methods used to compare study groups for primary methods outcome(s), including complex methods of correlated data | Generalized linear mixed effects models (with log link) and paired t-tests. |
|  | 11 | Statistical methods used for additional analyses, such as a subgroup analyses and adjusted analysis | Not applicable |
|  | 11 | Methods for imputing missing data, if used | None were used |
|  | 11 | Statistical software or programs used | R v3.6.1 |
| Participant flow | 12 | Flow of participants through each stage of the study: enrollment, assignment, allocation, and intervention exposure, follow-up, analysis (a diagram is strongly recommended) | Please see Figure 3 in the manuscript |
|  |  | Enrollment: the numbers of participants screened for eligibility, found to be eligible or not eligible, declined to be enrolled, and enrolled in the study | All parents who gave consent, all students who gave assent (and whose parents gave consent) were eligible. Five students and three parents declined to participate. |
|  |  | Assignment: the numbers of participants assigned to a study condition | Assignment was at the school level. |
|  |  | Allocation and intervention exposure: the number of participants assigned to each study condition and the number of participants who received each intervention | All 12 schools, 480 students, and 480 caregivers were in the intervention group. |
|  |  | Follow-up: the number of participants who completed the follow up or did not complete the follow-up (i.e., lost to follow-up), by study condition | 65% of student-caregiver pairs completed the follow-up. |
|  |  | Analysis: the number of participants included in or excluded from the main analysis, by study condition | The only participants that were excluded were students or caregivers who were interviewed, but their caregiver or student, respectively, was not interviewed during the same round of data. |
|  |  | Description of protocol deviations from study as planned, along with reasons | No deviations occurred. |
| Recruitment | 13 | Dates defining the periods of recruitment and follow-up | Recruitment occurred in June of 2016. Follow-up data collection occurred in September of 2017. |
| Baseline Data | 14 | Baseline demographic and clinical characteristics of participants in each study condition | Please see tables 2 and 3 in the main manuscript |
|  |  | Baseline characteristics for each study condition relevant to specific disease prevention research | Please see tables 2 and 3 in the main manuscript |
|  |  | Baseline comparisons of those lost to follow-up and those retained, overall and by study condition | Analysis available upon request |
|  |  | Comparison between study population at baseline and target population of interest | No data available on this topic |
| Baseline equivalence | 15 | Data on study group equivalence at baseline and statistical methods used to control for baseline differences | Please see tables 2 and 3 in the main manuscript |
| Numbers analyzed | 16 | Number of participants (denominator) included in each analysis for each study condition, particularly when the denominators change for different outcomes; statement of the results in absolute numbers when feasible | Please see table and figure titles, captions, and footnotes for this information |
|  |  | Indication of whether the analysis strategy was “intention to treat” or, if not, description of how non-compliers were treated in the analyses | All students and caregivers were included in the treatment group regardless of their participation in the curriculum. We used an “intention to treat” protocol. |
| Outcomes and estimation | 17 | For each primary and secondary outcome, a summary of results for each estimation study condition, and the estimated effect size and a confidence interval to indicate the precision | Please see tables and figures in the table |
|  |  | Inclusion of null and negative findings | Please see tables and figures in the table |
|  |  | Inclusion of results from testing pre-specified causal pathways through which the intervention was intended to operate, if any | All analyses were specified in the causal conceptual model included as Figure 1. |
| Ancillary analyses | 18 | Summary of other analyses performed, including subgroup or restricted analyses, indicating which are pre-specified or exploratory | Additional exploratory analyses were conducted around the impact of the learning object. Exploratory analyses on the potential for a “treatment on the treated” analysis on students reporting to attend WASH UP! sessions were conducted. Neither of these analyses were deemed worthy of inclusion in the manuscript. |
| Adverse events | 19 | Summary of all important adverse events or unintended effects in each study condition (including summary measures, effect size estimates, and confidence intervals) | No adverse or unintended events occurred in the study. |
| **DISCUSSION** |  |  |  |
| Interpretation | 20 | Interpretation of the results, taking into account study hypotheses, sources of potential bias, imprecision of measures, multiplicative analyses, and other limitations or weaknesses of the study | These are presented in the discussion section. We note areas where our results deviate from our conceptual model. |
|  |  | Discussion of results taking into account the mechanism by which the intervention was intended to work (causal pathways) or alternative mechanisms or explanations | The mechanism is described in detail in Figure 1, and this outline is followed throughout the paper. |
|  |  | Discussion of the success of and barriers to implementing the intervention, fidelity of implementation | Intervention fidelity is not explored in this paper due to the research team having no role in the implementation of the intervention. |
|  |  | Discussion of research, programmatic, or policy implications | Please see discussion section |
| Generalizability | 21 | Generalizability (external validity) of the trial findings, taking into account the study population, the characteristics of the intervention, length of follow-up, incentives, compliance rates, specific sites/settings involved in the study, and other contextual issues | Please see discussion section |
| Overall Evidence | 22 | General interpretation of the results in the context of current evidence and current theory | Please see discussion section |

## Table S9. Attrition Analysis for Caregivers: Comparison of demographic outcomes and key covariates between households that were lost to attrition after baseline and those who were interviewed at baseline and endline

|  | **Uses an improved drinking water source** | **Has dedicated place to wash their hands** | **Uses a shared or private latrine to defecate** | **Trusts the school as a source of information** | **Agrees that students should share what they learn in school with their parents at home** |
| --- | --- | --- | --- | --- | --- |
| Baseline only (N = 170) | 74% | 39% | 63% | 78% | 87% |
| Baseline and endline  (N = 310) | 75% | 31% | 57% | 79% | 86% |

## Table S10. Attrition Analysis for Students: Comparison of primary outcomes and key covariates between students that were lost to attrition after baseline and those who were interviewed at baseline and endline

|  | **Correctly identified taps and boreholes as safe sources of drinking water** | **Correctly identified surface water and shallow wells as unsafe sources of drinking water** | **Reported sharing a WASH message at home with their parents** | **Report feeling comfortable teaching their parent something new** |
| --- | --- | --- | --- | --- |
| Baseline only (N = 170) | 72% | 53% | 10% | 77% |
| Baseline and endline (N = 310) | 77% | 70%^***^ | 7% | 77% |

^***^ p < 0.001
